# Cystostomie percutanée à la pince de Kelly: indications, technique et résultats

**DOI:** 10.11604/pamj.2015.22.164.7280

**Published:** 2015-10-21

**Authors:** Ibrahima Diabaté, Bouréima Ouédraogo, Ibrahima Sow, Aliou Bâ

**Affiliations:** 1 Service d'Urologie, CHR Amadou Sakhir Mbaye, Louga, Sénégal; 2 Service de Chirurgie, CHR Amadou Sakhir Mbaye, Louga, Sénégal

**Keywords:** Cathétérisme vésical, cystostomie percutanée, pince de Kelly, sonde de Foley, rétention d´urine, bladder catheterization, percutaneous cystostomy, Kelly clamp, Foley catheter, urine retention

## Abstract

**Introduction:**

La dérivation urinaire sus-pubienne est pratiquée dans différentes circonstances. Cette étude vise à décrire la technique de cystostomie percutanée (CPC) pratiquée à l'aide d'une pince de Kelly pour la pose d'une sonde de Foley, à définir les indications de cette technique et à rapporter les résultats.

**Méthodes:**

Du 1er janvier 2005 au 31 décembre 2014, il a été réalisé 194 CPC à la pince de Kelly dans notre service, en urgence, sous anesthésie locale, chez des patients en rétention vésicale. Cette technique, dérivée de la cystostomie par ponction au trocart vise à placer dans la vessie une sonde de Foley après incision cutanée et aponévrotique (de 1 cm sur la ligne médiane, à 1,5 - 2 cm au-dessus de la symphyse pubienne) et la ponction vésicale à la pince de Kelly à travers cette incision.

**Résultats:**

Les 194 patients étaient tous de sexe masculin, âgés en moyenne de 50 ans ± 21 (extrêmes de 17 ans et 86 ans). Les pathologies à l'origine des rétentions vésicales étaient: les rétrécissements urétraux (n=119), les hypertrophies bénignes de la prostate (n=47), les cancers de prostate (n=21), les traumatismes de l'urètre (n=7). Tous les patients ont été opérés avec succès par cette méthode et les suites ont été simples. Le temps de réalisation était de 6 minutes ± 1. Les sondes de Foley mises en place étaient de charrière 16 (n=59), charrière 18 (n=116) et charrière 20 (n=19). La cicatrisation du trajet de la CPC après l'ablation de la sonde de Foley n'a posée aucun problème chez 146 patients suivis, les 48 autres ayant été perdus de vue.

**Conclusion:**

La CPC à la pince de Kelly est une technique simple, rapide et pas onéreuse. Ses indications sont les mêmes que pour toute CPC et elle représente une alternative à la cystostomie par chirurgie ouverte.

## Introduction

**Figure 1:**
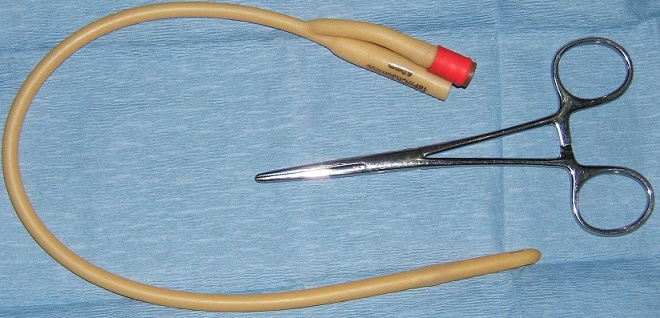
Sonde de Foley et pince de Kelly destinées à la CPC

La cystostomie percutanée (CPC) encore appelée cathétérisme vésical sus-pubien ou cystocathétérisme sus-pubien est une intervention dont le but est d'assurer le drainage des urines vésicales, de façon temporaire ou définitive. Elle est largement pratiquée et comporte des risques, heureusement rares [1–9]. L'un de ceux-ci est la chute du cathéter sus-pubien ou son obstruction, si ce dernier est dépourvu de ballonnet ou est de petit calibre [2, 4, 10]. D'où le choix de la cystostomie par chirurgie ouverte ou l'usage de kits [2, 8] permettant la pose d'une sonde de Foley. Par ailleurs, certaines techniques de CPC [1, 2, 6, 7, 9, 10], pratiquées sous contrôle échographique, fluoroscopique ou endoscopique permettent de placer d'emblée une sonde à ballonnet dans la vessie par voie percutanée. Toutefois, dans de nombreuses structures de soins en Afrique au sud du Sahara, la question du coût et de la disponibilité de ce matériel prêt à l'emploi, de ces équipements n'a encore pas été résolue. Le but de ce travail était de décrire la technique de CPC à l'aide d'une pince de Kelly pour l'insertion vésicale d'une sonde de Foley (Figure 1), de donner les indications et présenter les résultats en abordant au passage les autres techniques de CPC.

## Méthodes

Il s'agit d'une étude rétrospective et descriptive de 194 cas de CPC à la pince de Kelly, pratiquée dans notre institution entre le 1^er^ janvier 2005 et le 31 décembre 2014, selon une technique dérivée de la cystostomie par ponction au trocart. L'accord du comité éthique de la Commission médicale d’établissement de notre institution a été obtenu avant le début de pratique de cette technique. Toutes les interventions ont été pratiquées en urgence, sous anesthésie locale, par un urologue sénior après avoir obtenu le consentement libre et éclairé des patients. Ces CPC à la pince de Kelly étaient indiquées chez des patients en rétention vésicale complète avec un globe vésical palpable et qui présentaient une contre-indication ou un échec à la pose de sonde urétrale. Les contre-indications de cette technique étaient celles de toute cystostomie percutanée, en particulier les cicatrices de chirurgie pelvienne, les troubles de l'hémostase, les tumeurs vésicales, les rétentions vésicales par caillotage, l'absence de globe vésical.

**Matériels et équipements** Pour la réalisation de cette CPC, ont été utilisés: une sonde de Foley charrière 16, 18 ou 20; une pince de Kelly droite ou courbe (sans griffe) de 14 ou 16 centimètres de long; une seringue de 10 millilitres, de la lidocaïne, des compresses et un antiseptique.

**Figure 2:**
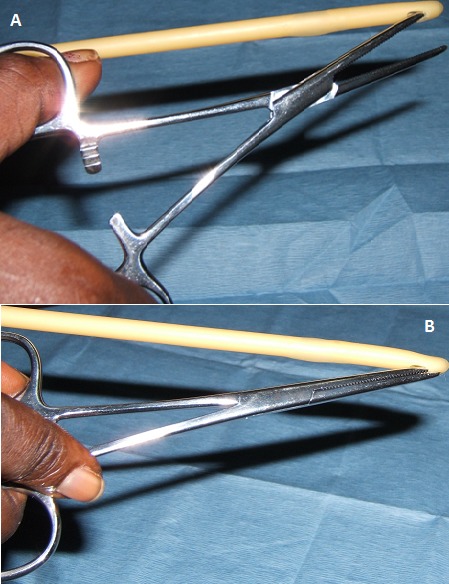
À titre démonstratif: A) Une branche de la pince de Kelly dans l’œillet de la sonde; B) Sonde armée après fermeture de la pince de Kelly

**Figure 3:**
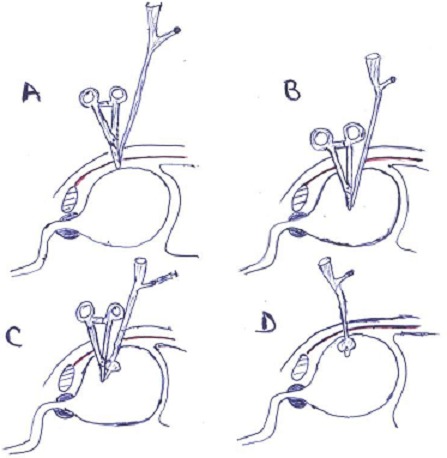
Étapes de la CPC à la pince de Kelly

**Figure 4:**
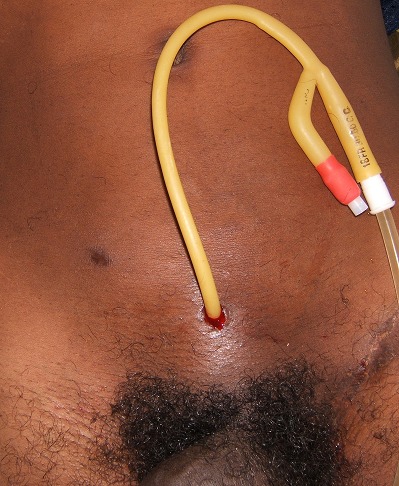
Aspect final de la CPC à la pince de Kelly

**Technique chirurgicale:** le patient en rétention vésicale est installé en décubitus dorsal; la désinfection de l'hypogastre est faite de même qu'une anesthésie locale à la lidocaïne par infiltration de la peau et des plans sous-cutanés, à 2 centimètres au-dessus de la symphyse pubienne et sur la ligne médiane; une incision cutanée et aponévrotique verticale de 1 cm est faite sur la ligne médiane à 1,5-2 cm au-dessus de la symphyse pubienne; la pince de Kelly est introduite à travers l'incision cutanée pour s'assurer que l'incision aponévrotique est effective et qu'il n'y a aucune résistance. La pince est ensuite retirée; une ponction vésicale test est faite à la seringue; la sonde de Foley, armée de la pince de Kelly (Figure 2 (A,B)) est introduite à travers l'incision cutanée et aponévrotique jusqu'au contact de la paroi antérieure de la vessie (Figure 3 (A); la ponction est faite (de la même manière qu'avec un trocart) en enfonçant la pince de Kelly dans la vessie distendue de façon perpendiculaire à la paroi abdominale (Figure 3 (B) et aussitôt le flux d'urine en direction de la poche à urine est constaté; la pince est maintenue enfoncée dans la vessie jusqu’à ce que l'aide ait gonflé le ballon de la sonde de Foley (Figure 3 (C). C'est seulement après que la pince est ouverte et retirée de l'incision. En guise de test, la sonde doit pouvoir être davantage enfoncée dans la vessie puis ramenée à sa position initiale sans résistance (Figure 4); ce test permet d’être sûr que le ballon de la sonde est bien gonflé dans la vessie. Un pansement est alors appliqué autour de la sonde.

## Résultats

Tous les 194 patients opérés selon la technique de CPC à la pince de Kelly étaient de sexe masculin. L’âge moyen était de 50 ans ± 21 avec des extrêmes de 17 ans et 86 ans. Les pathologies à l'origine des retentions d'urine étaient reparties en rétrécissements urétraux (n=119 cas), hypertrophie bénigne de la prostate (n=47 cas), cancer de prostate (n=21 cas), traumatismes de l'urètre (n=7 cas). Tous les 194 cas de CPC ont été réalisés avec succès. Les patients ont tous bien supporté l'intervention. Le temps de réalisation de l'acte était de 6 minutes ± 1. Les sondes de Foley utilisées étaient de différents calibres: charrière 16 (n=59), charrière 18 (n=116) et charrière 20 (n=19). Aucune complication n'a été observée. Les patients qui ont été suivis jusqu’à l'ablation de la sonde de Foley sus-pubienne étaient au nombre de 146. La durée moyenne du port de la sonde était de 21 jours ± 9. Chez 96 patients (rétrécissements urétraux et traumatismes de l'urètre), la fermeture du trajet percutané a été constatée 72 heures en moyenne après l'ablation de la sonde. Les patients opérés par taille vésicale (n=50) avec une incision à cheval sur le trajet de la CPC ont cicatrisé en 14 jours en moyenne. Nous avons perdu de vue 48 patients sur un total de 194.

## Discussion

La CPC, destinée au drainage des urines vésicales est particulièrement indiquée dans les cas d'impossibilité ou de contre-indication au cathétérisme urétral [6, 8], dans certaines résections endoscopiques [2] et dans la prévention de la distension vésicale pouvant compromettre les sutures après des interventions sur le bas appareil urinaire [9]. C'est une intervention particulièrement courante dans les services d'urgence [1, 3]. Dans une série de Diallo A.B et al [3], elle a été pratiquée dans 24,14% des cas de rétention d'urine vésicale. Quant à celle de Fall B et al [5] portant sur 1237 urgences urologiques, 331 interventions chirurgicales ont été pratiquées dont 59,8% étaient la CPC. Les taux de complications liées à cette intervention varient entre 1,6% et 2% selon les séries [1, 6]. Ceux sont en particulier les perforations de viscères [1, 2, 4, 6, 8, 9]: intestins, rectum, utérus, vagin; les complications hémorragiques [4, 6, 8]: hématurie par blessure prostatique, hématome péri-vésical; les chutes de sonde [2, 4]. Dans notre série, nous n'avons rencontré aucune complication de cette nature en raison de notre extrême vigilance dans la pratique de ce geste chirurgical et la sélection des cas. D'autres méthodes ou techniques et différents kits de ponction sus-pubienne existent [1, 2, 6–9]. Chiou R.K et al [2] ainsi que d'autres auteurs [4, 7, 8] ont proposé des méthodes de CPC basées sur la dilatation progressive du trajet percutané permettant d'insérer dans la vessie une sonde de Foley. Bien que sûres, ces méthodes nous paraissent complexes et peuvent prendre dans leur exécution beaucoup de temps surtout si elles s'accompagnent de l’échographie [1, 6] ou de la fluoroscopie [7, 8]. Ceci pourrait augmenter l'angoisse d'un patient sous anesthésie locale, qui attend d’être soulagé d'une rétention d'urine. Sawant A.S et al [9], tout comme Lawrentschuk N et al [6] ont utilisé la voie endoscopique urétro-vésicale pour les CPC, mais cela suppose un urètre indemne de toute lésion contre-indiquant une exploration endoscopique. A cela, s'ajoute pour toutes ces techniques la nécessité de disposer de kits, de matériel d'imagerie et d'endoscopie sans oublier leur coût parfois élevé pour des populations démunies. Plus récemment, Okorie [10] proposait une CPC à moindre coût comme la nôtre, avec pose d'une sonde de Foley après ponction vésicale à l'aide d'un scalpel. Les autres avantages de la technique que nous avons pratiquée sont: la simplicité de la technique, la facilité et la rapidité d'exécution avec un matériel presque partout disponible. Pratiquée sous anesthésie locale en salle d'urgence, elle a permis à l'aide d'une pince de Kelly de placer d'emblée dans la vessie, en une seule ponction une sonde de Foley. L'utilisation d'une sonde de charrière 18 ou 20 a été une alternative intéressante à la cystostomie par chirurgie ouverte dont on connaît les exigences: une anesthésie générale ou locorégionale, une incision large, une durée d'hospitalisation plus longue. Bien que les 194 CPC n'aient été pratiquées que chez des patients de sexe masculin, rien ne contre-indique cette technique chez des patients de sexe féminin. Ses risques et complications sont les mêmes que pour toute CPC, exceptée la chute de la sonde car celle-ci est maintenue dans la vessie par le ballonnet gonflé. L'inconvénient de cette technique est d'avoir la vessie pleine et distendue avant toute ponction à la pince de Kelly, alors que la méthode endoscopique à l'urétrotome proposée par Sawant A.S et al [9] ne nécessite pas de réplétion vésicale. Aucune complication n'a été rapportée dans cette série et les résultats peuvent être jugés bons. Nous pensons qu'elle est à la portée de tout médecin ayant l'habitude des autres techniques de CPC.

## Conclusion

La CPC à la pince de Kelly que nous pratiquons dans notre institution en situation d'urgence sur une vessie distendue, est une technique simple, rapide et absolument pas onéreuse adapté à un contexte de sous équipement. Elle ne nous a valu aucune complication et demeure une alternative à la cystostomie par chirurgie ouverte.

## References

[1] Aguilera PA, Choi T, Durham BA (2004). Ultrasound-guided suprapubic cystostomy catheter placement in the emergency department. J Emerg Med..

[2] Chiou RK, Morton JJ, Engelsgjerd JS, Mays S (1995). Placement of large suprapubic tube using peel-away introducer. J Urol..

[3] Diallo AB, Bah I, Diallo TMO (2010). Le profil des urgences urologiques au CHU de Conakry, Guinée. Prog Urol..

[4] Dogra PN, Goel R (2004). Complication of percutaneous suprapubic cystostomy. Int Urol Nephrol..

[5] Fall B, Diao B, Fall PA (2008). Les urgences urologiques en milieu hospitalier universitaire à Dakar: aspects épidémiologiques, cliniques, thérapeutiques. Prog Urol..

[6] Lawrentschuk N, Lee D, Marriott P (2003). Suprapubic stab cystostomy: a safer technique. Urology..

[7] Lee MJ, Papanicolaou N, Nocks BN (1993). Fluoroscopically guided percutaneous suprapubic cystostomy for long term bladder drainage: an alternative to surgical cystostomy. Radiology..

[8] Papanicolaou N, Pfister RC, Nocks BN (1989). Percutaneous, large-bore, suprapubic cystostomy: technique and results. AJR Am J Roentgenol..

[9] Sawant AS, Patwardhan SK, Attar MI (2009). Suprapubic cystostomy using optical urethrotome in female patients. J Endourol..

[10] Okorie CO (2014). Simplified percutaneous large bore suprapubic cystostomy for acute urinary retention - A cost saving procedure. Afr J Urol..

